# Extracts of *Thesium chinense* inhibit SARS-CoV-2 and inflammation *in vitro*

**DOI:** 10.1080/13880209.2023.2253841

**Published:** 2023-09-07

**Authors:** Juncheng Ma, Juanru Wei, Gang Chen, Xiaowei Yan, Hechun Sun, Ning Li

**Affiliations:** Inflammation and Immune Mediated Diseases Laboratory of Anhui Province, School of Pharmacy, Anhui Medical University, Hefei, China

**Keywords:** *Thesium chinense* Turcz, SARS-CoV-2, COVID-19, anti-inflammatory

## Abstract

**Context:**

The severe acute respiratory syndrome coronavirus 2 (SARS-CoV-2) is still spreading rapidly. Relevant research based on the antiviral effects of *Thesium chinense* Turcz (Santalaceae) was not found.

**Objective:**

To investigate the antiviral and anti-inflammatory effects of extracts of *T. chinense*.

**Materials and methods:**

To investigate the anti-entry and replication effect of the ethanol extract of *T. chinense* (drug concentration 80, 160, 320, 640, 960 μg/mL) against the SARS-CoV-2. Remdesivir (20.74 μM) was used as positive control, and Vero cells were used as host cells to detect the expression level of nucleocapsid protein (NP) in the virus by real-time quantitative polymerase chain reaction (RT-PCR) and Western blotting. RAW264.7 cells were used as an anti-inflammatory experimental model under lipopolysaccharide (LPS) induction, and the expression levels of tumor necrosis factor-alpha (TNF-α) and interleukin-6 (IL-6) were detected by enzyme-linked immunosorbent assay (ELISA).

**Results:**

The ethanol extract of *T. chinense* significantly inhibited the replication (half maximal effective concentration, EC_50_: 259.3 μg/mL) and entry (EC_50_: 359.1 μg/mL) of SARS-CoV-2 into Vero cells, and significantly reduced the levels of IL-6 and TNF-α produced by LPS-stimulated RAW264.7 cells. Petroleum ether (EC_50_: 163.6 μg/mL), ethyl acetate (EC_50_: 22.92 μg/mL) and *n*-butanol (EC_50_: 56.8 μg/mL) extracts showed weak inhibition of SARS-CoV-2 replication in Vero cells, and reduced the levels of IL-6 and TNF-α produced by LPS-stimulated RAW264.7 cells.

**Conclusion:**

*T. chinense* can be a potential candidate to fight SARS-CoV-2, and is becoming a traditional Chinese medicine candidate for treating COVID-19.

## Introduction

Coronaviruses are a class of positive single-stranded RNA viruses with encapsulation, which evolves rapidly due to their high nucleotide substitution and recombination rates, and can infect a variety of mammals including humans (Fung and Liu 2019). Since the beginning of the twenty first century, coronavirus has appeared periodically all over the world, such as severe acute respiratory syndrome coronavirus (SARS-CoV), Middle East respiratory syndrome coronavirus (MERS-CoV), and currently circulating SARS-CoV-2 that caused coronavirus disease 2019 (COVID-19). They are all related to the major outbreak of human fatal pneumonia (Kirtipal et al. 2020). With extremely high pathogenicity and infectivity, COVID-19 has rapidly spread to all parts of the world and caused extremely serious respiratory diseases, becoming a new public health problem in the twenty first century. By April 22, 2023, about 686 million people had been infected with SARS-CoV-2, distributed in more than 200 countries and regions around the world, and the cumulative death cases exceeded 6.85 million. Therefore, the prevention and treatment of novel coronavirus strains have become a major scientific challenge in the field of global health today. The research and development of drugs (Wang et al. 2022; Wang and Yang 2022) to effectively prevent and treat COVID-19 has become a major strategic and social demand. There is an urgent need to discover and provide safe, original anti-novel coronavirus candidate drugs (Wang et al. 2022) with clear activity, and a high drug completion rate.

Conventional antiviral drugs, of which nucleoside analogs are the most common, include favipiravir, ribavirin, and remdesivir. Drugs enter virus-infected cells and form adenine or guanine nucleoside analogs that competitively inhibit key enzymes of viral RNA and protein synthesis, such as viral RNA polymerase, thus interfering with and blocking viral replication and transmission (De Clercq 2019). Although all of them can inhibit viral RNA replication, there are certain limitations in the safety and effectiveness of clinical medication. The toxic side effects of ribavirin monotherapy exceed the potential benefits (Mo and Fisher 2016), and the effect of large doses of ribavirin on SARS-CoV may lead to adverse events (Muller et al. 2007). Fapiravir clinical use showed a trend towards improved survival in patients treated with the *Ebola* virus, but the effect of treatment was not statistically significant (Kerber et al. 2019). Remdesivir has no significant clinical or antiviral effect in patients with severe COVID-19 but does show clinical improvement in patients treated early, which still needs to be confirmed in a larger number of studies (Wang et al. 2020). There have been studies showing very low levels of Remdesivir resistance (Focosi et al. 2022), but there is also literature pointing to the emergence of resistant strains in clinical cases (Gandhi et al. 2022). However, remdesivir significantly reduces the viral load at the cellular and animal levels (Pruijssers et al. 2020; Williamson et al. 2020). Although scholars have different opinions on the clinical use of Remdesivir, in our preliminary experiment results, Remdesivir showed better anti-SARS-CoV-2 effect, so we used Remdesivir as our positive control. Of course, there are also natural plants with better antiviral effects (Yang and Wang 2021). For example, glycyrrhizic acid, the active ingredient in licorice root, can significantly inhibit the replication of the SARS-CoV and inhibit virus adsorption and penetration (Cinatl et al. 2003). The ethanolic extract of *Scutellaria baicalensis* Georgi (Labiatae) and its major component baicalein inhibited the activity of SARS-CoV-2 and its 3 C-like protease *in vitro*, and both inhibited the replication and entry of SARS-CoV-2 in Vero cells (Liu et al. 2021). Therefore, we can continue trying to discover natural plants with an anti-coronavirus effect.

*Thesium chinense* Turcz (Santalaceae) is the dried whole plant. It has antibacterial and anti-inflammatory effects, reduce body temperature, detoxifies, and is a broad-spectrum antibacterial Chinese herbal medicine. Because of curative and rapid effects, known as ‘natural antibiotics’, clinical antibiotics are often used to treat mastitis, tonsillitis, pharyngitis, all kinds of pneumonia and upper respiratory tract infections (Parveen et al. 2007). Pharmacological studies have shown that *T. chinense* has good effects in anti-inflammatory (Sun et al. 2019), antioxidation (Shao et al. 2020), antibacterial (Liu et al. 2018), analgesia and other aspects, and has a broad application prospects. At present, there is no literature report on the anti-SARS-CoV-2 of *T. chinense*, but kaempferol, as a monomeric compound isolated from *T. chinense*, can inhibit the replication of human coronavirus (Cheng and Wong 1996). Kaempferol has been found to have a strong affinity for the S protein and angiotensinase 2 (ACE2) of SARS-CoV-2 by molecular docking (Yang et al. 2020), and it is also one of the main active ingredients in commonly used Chinese herbal medicines for the prevention and treatment of COVID-19 (Li et al. 2020). Bacterial co-infection is a common complication of many viral respiratory tract infections. Some studies have found that mild symptoms and long-term hospitalized COVID-19 patients are more likely to cause bacterial co-infection (Westblade et al. 2021; Davies-Bolorunduro et al. 2022). *T. chinense* has an excellent effect on treating inflammation, microbial infection and upper respiratory tract disease (Li et al. 2021), and has an obvious inhibitory effect on a variety of bacteria (Liu et al. 2006). Therefore, *T. chinense* can inhibit a variety of bacterial infections, reduce the severity of COVID-19 and avoid complications. This indicates that *T. chinense* may have a unique role in the treatment of diseases caused by SARS-CoV-2.

Infection with coronavirus can lead to the release of a large number of proinflammatory cytokines and a cytokine storm caused by the maladjustment of body release, which caused serious damage to host tissues and organs by stimulating the death of inflammatory cells (Zheng et al. 2021). The cytokine storm caused by SARS-CoV-2 is considered to be the main cause of disease progression (Pum et al. 2021). In clinical studies, serum levels of IL-6 and TNF-α are independent and significant predictors of disease severity and death in critically ill patients with COVID-19 (Del Valle et al. 2020). Therefore, we wanted to further study the anti-inflammatory effects of different polar parts of *T. chinense*, using LPS to stimulate RAW264.7 cells to produce inflammatory factors as a model, and using IL-6 and TNF-α as detection indices. The anti-inflammatory effects of extracts from different polar parts of *T. chinense* against SARS-CoV-2 were studied *in vitro*.

The SARS-CoV-2 is still spreading and mutating around the world, but the current traditional anti-coronavirus drugs still have certain limitations. Traditional Chinese medicine has rich experience in the prevention and control of plague. Due to its complex composition, it can exert the overall effect through multi-level, multi-target and multi-pathway, and can improve symptoms and reduce mortality and recurrence rate. Therefore, this experiment explores the antiviral and anti-inflammatory effects of *T. chinense*.

## Materials and methods

### Sample preparation

The whole dry herb of *T. chinense* was collected from Xiangyang City, Hubei Province, P. R. China in May 2019. The species was identified by Prof. Kai-Jin Wang at the School of Life Sciences, Anhui University, and a voucher number (No. 20190927) was deposited in the School of Pharmacy, Anhui Medical University. A total of 230 g of *T. chinense* was added to 2 L of 95% ethanol solution for 12 h and ultrasonicated at 50 °C for 3 h. Then, the filtrate was collected, and the above steps were repeated with 85% and 70% ethanol solution for the filter residue. Half filtrate was concentrated and then freeze-dried (denoted BRY). The other half filtrate was extracted with 1 L petroleum ether at 20 °C for 30 min, 4 times in total, and then the organic phase was collected and concentrated (denoted BS1). Then, the above steps were repeated with ethyl acetate (denoted BY2) and *n*-butanol (denoted BZ3) (El-Hilaly et al. 2021). The filtrate residue after ethanol extraction was extracted twice at 80 °C for 3 h in 2 L distilled water by ultrasound and then filtered. The filtrate was concentrated and then freeze-dried (denoted BRS). All dry extracts were completely dissolved in dimethyl sulfoxide (DMSO) to obtain 160 mg/mL mother liquor. Remdesivir (denoted RDV) was purchased from Shanghai YuanYe Biotechnology Co., Ltd. Remdesivir was dissolved in DMSO to obtain 1 mg/mL mother liquor, and all samples were stored at 20 °C before use. Ethanol, petroleum ether, ethyl acetate, *n*-butanol and other organic solvents were purchased from Shanghai Titan Scientific Co., Ltd.

### Cell lines and virus

African green monkey kidney cells (Vero) were treated with Dulbecco’s Modified Eagle Medium supplemented with 10% fetal bovine serum, 2% l-glutamine, and 1% penicillin/streptomycin (DMEM, Gibco, USA). Mouse monocyte-macrophage leukemia cells (RAW264.7) were treated with Dulbecco’s Modified Eagle Medium supplemented with 10% fetal bovine serum, 2% l-glutamine, and 1% penicillin/streptomycin (DMEM, BasalMedia, Shanghai). The cells were cultured at 37 °C and 5% CO_2_. Both cell lines were obtained from ATCC. SARS-CoV-2 (a virus strain isolated from the laboratory of Anhui Provincial Centers for Disease Control and Prevention in Suzhou Patient 005 in 2020) proliferated in Vero cells, and the virus titer was measured by 50% tissue culture infection dose (TCID_50_) using the Karber method based on the cytopathic effect. SARS-CoV-2 infection experiments were conducted in the Biosafety Level 3 (BSL-3) laboratory, and other infection experiments were conducted in the BSL-2 laboratory.

### Cytotoxicity assay

The methyl thiazolyl tetrazolium (MTT, Biofroxx, Germany) method was used to detect the cytotoxic effects of different polar extracts (BRY, BS1, BY2, BZ3, BRS) and positive drugs (RDV) on Vero cells. Single-layer growth Vero cells were incubated with the extracts at the specified concentration on a 96-well plate at 37 °C with 5% CO_2_ for 24 h, then 20 μL MTT of 5 mg/mL was added to each well. The incubation was continued at 37 °C and 5% CO_2_ for 4 h. The cell supernatant was discarded, and 100 μL DMSO was added to each well to evenly oscillate (Attallah et al. 2021). The light absorption value of each well was measured and recorded with a microplate reader at a wavelength of 570 nm. The cell inhibition rate and half maximal cytotoxicity (CC_50_) were calculated using GraphPad Prism 8.0 software. The cytotoxic effects of different polar extracts on RAW264.7 cells were also determined by the above methods and operations, and the half maximal inhibitory concentration (IC_50_) was calculated.

### Antiviral activity assay

Vero cells were inoculated in a 96-well plate at a density of 1.5 × 10^4^ cells/well and incubated at 37 °C and 5% CO_2_ for 24 h. To explore the antiviral replication effect, cells were infected with 30 TCID_50_ SARS-CoV-2 (TCID_50_ was 10^−3.5^), and the infected volume was 20 μL per well for 1.5 h. The supernatant was removed, and 100 μL of virus culture medium containing preconfigured drugs at different concentrations was added. After 48 h, the virus cells were frozen, thawed and collected. To explore the antiviral entry effect, Vero cells were pretreated with a virus culture medium with different concentrations of drugs for 3.5 h. Then, the SARS-CoV-2 was added and coincubated for 1.5 h, after which the virus and drugs were washed away. After 48 h, freeze-thaw virus cells were collected for RT–PCR detection.

### Anti-inflammatory activity assay

RAW264.7 cells were inoculated in a 24-well plate at a density of 5 × 10^4^ cells/well, and incubated at 37 °C and 5% CO_2_ for 24 h. In the sample solution group, the culture medium was changed to medium containing LPS (1 μg/mL, Sigma, USA) and different concentrations of drugs to induce RAW264.7 differentiation. In the blank control group, the culture medium was changed to fresh medium, and in the model group, the culture medium was changed to medium containing LPS (1 μg/mL) to induce RAW264.7 differentiation. The expression levels of TNF-α and IL-6 were detected by ELISA.

### Western blot

Vero cells were inoculated in 6-well plates at a density of 2.5 × 10^6^ cells/well, and cultured at 37 °C and 5% CO_2_ for 24 h. To explore the antiviral replication effect, SARS-CoV-2 was first added to Vero cells for 1.5 h, and the infected volume of each well was 400 μL. The virus was washed, and then sample solutions of different concentrations were added to the infected Vero cells and incubated for 24 h. RIPA (Beyotime, Shanghai, China) cell lysate (200 μL) containing phosphatase inhibitor (Beyotime, Shanghai, China) and protease inhibitor (Beyotime, Shanghai, China) was added to each well and placed on ice for 30 min to extract cell proteins. To explore the antiviral entry effect, Vero cells were pretreated with a virus culture medium with different concentrations of drugs for 3.5 h. Then SARS-CoV-2 was added and coincubated for 1.5 h, after which the virus and drugs were washed away. Cell protein was extracted after 24 h. The cell lysate was centrifuged at 12000 rpm at 4 °C for 20 min. After centrifugation, the supernatant was taken and an equal volume of SDS–PAGE sample loading buffer (Beyotime) was added and then heated at 100 °C for 10 min. They were separated by 10% SDS–PAGE, transferred to a polyvinylidene fluoride (PVDF, Merck Millipore Ltd., Ireland) membrane, sealed with 5% skim milk, washed with TBST 3 times, 15 min each time, and incubated overnight with SARS-CoV-2 nucleocapsid antibody (GTX632269, GeneTex, North America) and antibodies against GAPDH (AF7021, Affinity, USA) at 4 °C. TBST was used for washing 3 times, 15 min each, and the membrane was incubated with horseradish peroxidase (HRP) labeled secondary antibody (goat anti-rabbit IgG HRP and goat anti-mouse IgG, Affinity, USA). Images were obtained by the ECL (Glpbio, Montclair, USA) chemiluminescence method and quantified with ImageJ software (Bio-Rad, USA).

### RNA extraction and RT–PCR

For the antiviral experiment, the copy number of viral nucleic acid was detected by TaqMan probe RT-PCR. Viral RNA was extracted from the supernatant of cells using an automated nucleic acid extraction system (Tianlong, Hangzhou, China) and reverse-transcribed using 4 × TaqMan® FastVIRus1-StepMastermix (Thermo Fisher, USA). The full-length SARS-CoV-2 N gene was synthesized and cloned into pcDNA3.1. A standard curve was prepared by measuring the copy number of the plasmid diluted (3 × 10^2^ - 3 × 10^6^ copies). Primer sequences are as follows:

N: F-GGGGAACTTCTCCTGCTAGAAT; R-CAGACATTTTGCTCTCAAGCTG and probe: 5′-FAM-TTGCTGCTGCTTGACAGATT-TAMRA-3′.

### ELISA

For anti-inflammatory experiments, the expression levels of TNF-α and IL-6 were detected by ELIASA (ProteinTech, China). Standard curves were prepared according to the kit requirements. When adding samples, according to the preliminary experimental results, the IL-6 sample well was diluted 3 times, and the TNF-α sample well was diluted 20 times. After washing, detection antibodies were added and incubated at 37 °C for 1 h. After washing, streptavidin labeled with HRP was added, incubated at 37 °C for 40 min, and then washed again. For color development, TMB color development solution was added to each well for 15–20 min at 37 °C. When terminating, terminating solution was added to each well, and the blue will turn yellow. The optical density (OD) of each well was measured at 450 nm with a microplate reader at 630 nm as the correction wavelength. For data analysis, the OD value of each standard and sample was subtracted from the blank-hole OD value. With the concentration of the standard substance as the abscissa and the OD value as the ordinate, the professional software ELISACalc was used for four-parameter fitting (4-PL). The fitting concentration was calculated from the standard curve according to the OD value of the sample, and then the measured concentration of the sample was obtained by multiplying the dilution factor.

## Results

### Antiviral effect of extracts from different polar parts of T. chinense against SARS-CoV-2

**Figure 1.**
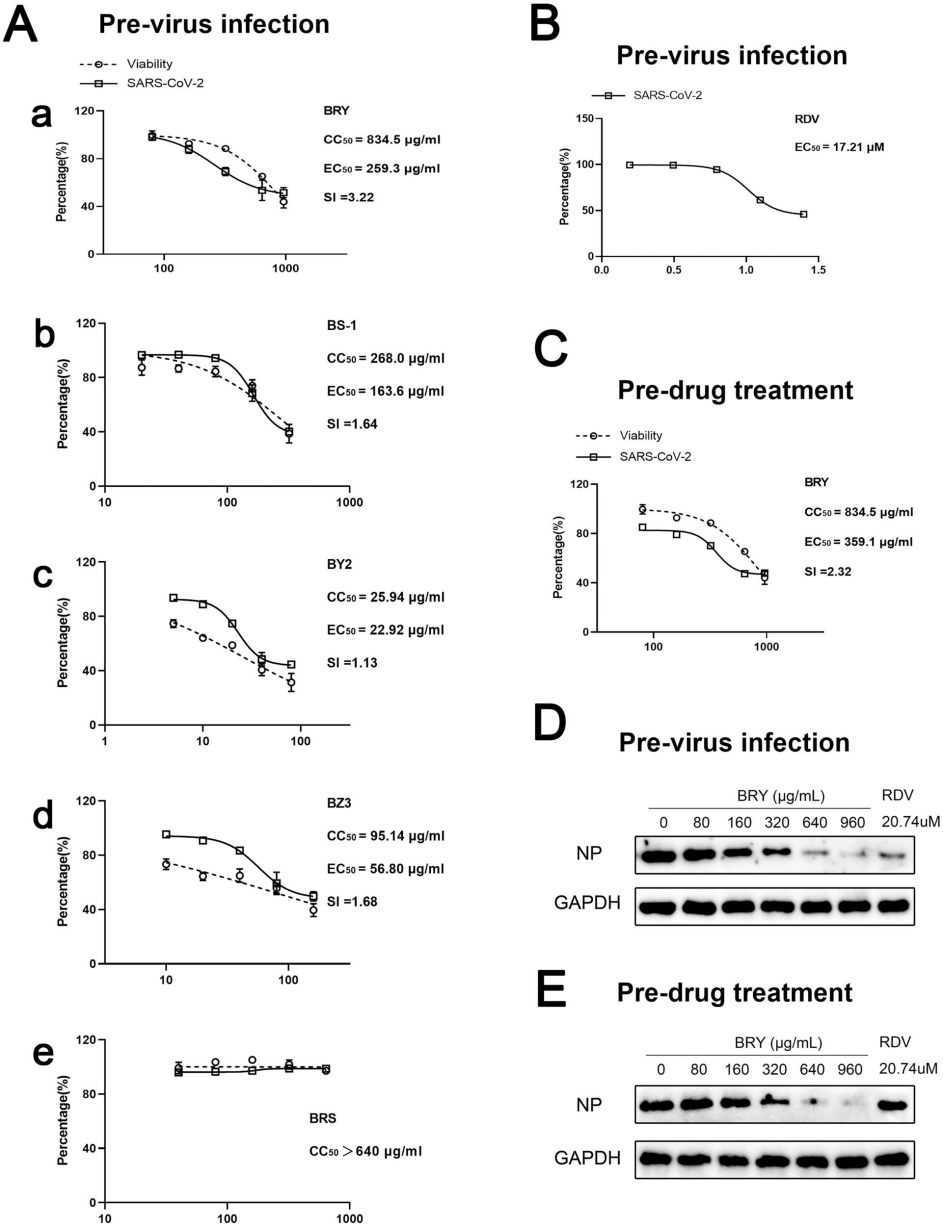
Antiviral effect of extracts from different polar parts of *T. chinense* against SARS-CoV-2. A. Effects of ethanol extract of *T. chinense* (a), petroleum ether extract of *T. chinense* (b), ethyl acetate extract of *T. chinense* (c), *n*-butanol extract of *T. chinense* (d), and water extract of *T. chinense* (e) on Vero cell viability (CC_50_) and antiviral replication in Vero cells (EC_50_). B. positive drug remdesivir for antiviral replication in Vero cells (EC_50_). C. Antiviral entry effect of ethanol extract of *T. chinense* in Vero cells. D. Western blot analysis of nucleocapsid levels in Vero cells at virus replication. E. Western blot analysis of virus at nucleocapsid level during entry into Vero cells.

We first investigated the effects of BRY, BS1, BY2, BZ3 and BRS on Vero cell viability. BRS had no significant effect on Vero cell viability when the drug concentration was 640 μg/mL (Figure 1Ae). The inhibitory effect of BRY, BS1, BY2 and BZ3 on Vero cell viability increased with increasing drug concentration, and their CC_50_ values were 834.5 μg/mL BRY, 268.0 μg/mL BS1, 25.94 μg/mL BY2, and 95.14 μg/mL BZ3 (Figure 1Aa–d). In the study of antiviral replication effects, BRY showed an extremely significant antiviral replication effect with an EC_50_ at 259.3 μg/mL, the cytotoxicity was weak, accompanying the therapeutic index (SI) of 3.22 (Figure 1Aa). BS1 (EC_50_: 163.6 μg/mL, SI: 1.64, Figure 1Ab) and BZ3 (EC_50_: 56.8 μg/mL, SI: 1.68, Figure 1Ad) also showed anti-replicating effects. BY2 (EC_50_: 22.92 μg/mL, SI: 1.13, Figure 1Ac) had an antiviral replication effect at a certain concentration; however, it also significantly inhibited cell viability at the same concentration, causing a low level of SI. BRS showed no effect against replicating viruses (Figure 1Ae). Further study showed that BRY also had a significant antiviral entry effect with an EC_50_ of 359.1 μg/mL and an SI of 2.32 (Figure 1C). Based on RT–PCR, we further investigated the specific NP levels in the BRY replication and entry stages of SARS-CoV-2 and found that the NP content decreased with the increase of BRY drug concentration, consistent with the RT–PCR results (Figure 1D,E). However, the positive drug RDV (EC_50_: 17.21 μΜ, Figure 1B) has no antiviral entry effect, because of its nucleoside analog that only inhibits virus replication and does not affect virus entry.

### Anti-inflammatory effect of extracts from different polar parts of T. chinense

**Figure 2.**
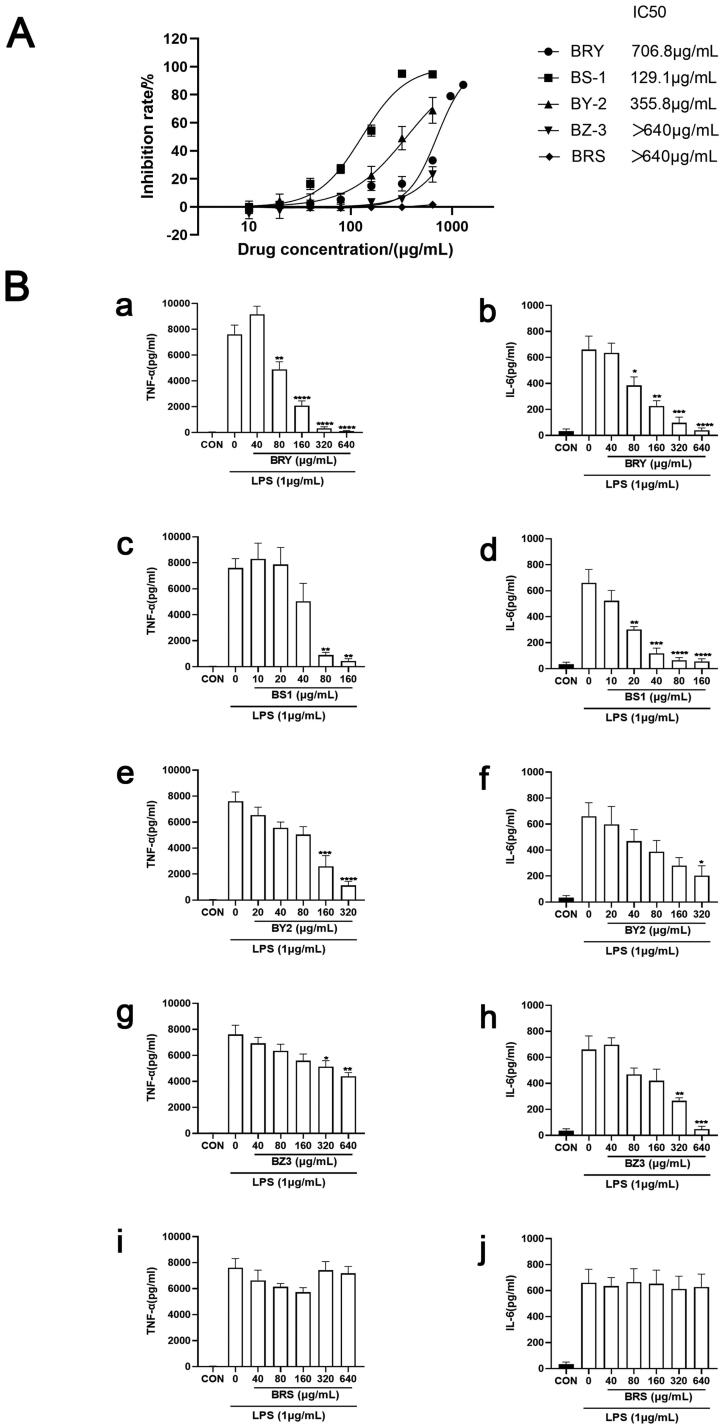
Anti-inflammatory effect of extracts from different polar parts of *T. chinense*. A. ethanol extract of *T. chinense*, petroleum ether extract of *T. chinense*, ethyl acetate extract of *T. chinense*, *n*-butanol extract of *T. chinense* and water extract of *T. chinense* inhibitory effect on RAW264.7 cells (IC_50_). B. Effects of ethanol extract of *T. chinense* (a, b), petroleum ether extract of *T. chinense* (c, d), ethyl acetate extract of *T. chinense* (e, f), *n*-butanol extract of *T. chinense* (g, h) and water extract of *T. chinense* (i, j) on the expression levels of TNF-α and IL-6 in RAW264.7 cells stimulated by LPS. Compared with virus control group, * *p* < 0.05; ** *p* < 0.01; *** *p* < 0.001; **** *p* < 0.0001.

The anti-inflammatory effects of different polar parts of *T. chinense* were investigated, using LPS (1 μg/mL) to stimulate RAW264.7 cells to produce inflammatory factors IL-6 and TNF- by ELISA kit. The results showed that BRS and BZ3 had no obvious effect on the viability of RAW264.7 cells at a concentration of 640 μg/mL, while BY2, BZ3 and BRS had obvious inhibitory effects on the viability of RAW264.7 cells. The IC_50_ values were 706.8 μg/mL for BRY, 129.1 μg/mL for BS1, and 355.8 μg/mL for BY2 (Figure 2A). In the inflammatory experiment model group, the content of IL-6 and TNF-α in the medium was significantly increased after LPS stimulation. BRY showed obvious dose dependence from 80 μg/mL to 640 μg/mL. With increasing drug concentration, the release of TNF-α and IL-6 was significantly inhibited. BRY significantly inhibited the release of TNF-α and IL-6 at a concentration of 640 μg/mL compared with the virus group (*p* < 0.0001, Figure 2Ba,b). BS1 significantly inhibited the release of IL-6 (*p* < 0.0001) and TNF-α (*p* < 0.01) at concentrations of 80 μg/mL and 160 μg/mL in a dose-dependent manner compared with the virus group (Figure 2Bc,d). BY2 showed obvious drug dependence at 20 μg/mL and significantly inhibited the release of TNF-α and IL-6 with increasing drug concentration. Compared with the virus group (*p* < 0.0001, Figure 2Be,f), BZ3 significantly inhibited the release of IL-6 at a concentration of 640 μg/mL (*p* < 0.0001), and the inhibition of TNF-α was weaker (*p* < 0.01, Figure 2Bg,h). BRS had a weak effect on TNF-α release at drug concentrations of 40 μg/mL and 160 μg/mL, with statistical significance. Moreover, BRS did not inhibit the release of IL-6 (Figure 2 Bi,j).

## Discussion

SARS-CoV-2 is still spreading worldwide, and research and development of related drugs are crucial for epidemic prevention and control. Traditional Chinese medicine has been used for fighting against diseases for thousands of years with rich theoretical and practical experience. Natural products are stable in the human gastrointestinal tract, increasing their bioavailability, and they have a long track record in the treatment of respiratory infections. Many natural products have been approved as drugs and are generally safe, so they are often used in combination therapy. In the face of this epidemic, traditional Chinese medicine has also played a significant role in treating COVID-19 (Wang and Yang 2021). It is the active intervention of traditional Chinese medicine that further improved clinical treatment efficacy (Chen and Chen 2020). Therefore, we focused on natural medicines, and tried to find a traditional Chinese medicine with anti-coronavirus effects. According to ancient books and literature review, we selected *T. chinense*, known as a ‘natural antibiotic’, to explore the effects of extracts from different polar parts of *T. chinense* on novel coronaviruses and their anti-inflammatory effects.

The replication cycle of SARS-CoV-2 includes adsorption, entry, replication, assembly and secretion. In the replication cycle of SARS-CoV-2, NP plays a key role in the virus replication, assembly and immune regulation (Peng et al. 2020). It is an important indicator of progeny virus formation. Therefore, NP was chosen as the research index in our experiment. In our study of the antiviral entry experiment, the SARS-CoV-2 was first added to Vero cells for 1.5 h to wash away the virus, and then different concentrations of extracts of different polar parts of *T. chinense* were added to treat infected Vero cells. The results showed that the ethanol, petroleum ether, ethyl acetate and *n*-butanol extracts could inhibit the replication of the virus, and the ethanol extracts were better than the other extracts. However, petroleum ether, ethyl acetate, and *n*-butanol extracts have antiviral activity but also present strong toxicity. To further explore the stage of antiviral replication, Vero cells were pretreated with different concentrations of *T. chinense* ethanol extract for 3.5 h, incubated with 2019-nCoV virus for 1.5 h, and then washed away the virus and drug. It was found that the ethanol extracts of *T. chinense* could inhibit the entry of the virus. According to the results of the RT-PCR and WB experiments, we can intuitively see that the amount of NP decreases significantly with increasing drug concentration, which proves that the ethanol extract of *T. chinense* can further inhibit virus replication in cells by inhibiting the generation of NP. Kaempferol, as a monomeric compound isolated from *T. chinense*, has been found to have a strong affinity for ACE2 of SARS-CoV-2 (Pan et al. 2020). The glycosidic derivatives of kaempferol have also been proven to be inhibitors of virus release, and the combination with 3CLpro of SARS-CoV-2 has the most advantages (Chen et al. 2021; Liao et al. 2021), which gives us the inspiration to explore the antiviral mechanism of *T. chinense* in future studies.

Infection with SARS-CoV-2 can lead to the release of a large number of proinflammatory cytokines (Zheng et al. 2021), and the serum levels of IL-6 and TNF-α are significantly increased in critically ill patients with COVID-19, which is important factors affecting the severity of disease and death of patients. Both IL-6 and TNF-α are pleiotropic cytokines that play important roles in the pathogenesis of most acute inflammatory diseases (Kalliolias and Ivashkiv 2016). Many IL-6 inhibitors and TNF-α inhibitors have been used in clinical practice (Dorner and Kay 2015; Rossi et al. 2015), and more studies have shown that IL-6 inhibitors can effectively treat COVID-19 (Xu et al. 2020). Therefore, we studied the anti-inflammatory effects of different polarities of *T. chinense* using LPS-stimulated RAW264.7 cells as a model of inflammation, and the expression levels of TNF-α and IL-6 were detected by ELISA. In the model group, the content of IL-6 and TNF-α in the medium was significantly increased after LPS stimulation. BRY, BS1, BY2 and BZ3 showed significant concentration dependence within specific drug concentrations, and gradually decreased the expression levels of IL-6 and TNF-α induced by LPS with increasing concentrations. BRY showed the best anti-inflammatory effect. Compared with the virus group, BRY significantly inhibited the release of TNF-α and IL-6 at drug concentrations of 320 μg/mL and 640 μg/mL (*p* < 0.0001). BRS had no effect on LPS-induced IL-6 expression levels but had a slight inhibitory effect on LPS-induced TNF-α expression levels at the low concentration of 160 μg/mL. In previous studies, ethyl acetate extract of *T. chinense* showed a significant anti-inflammatory effect on xylene-induced ear edema (Parveen et al. 2007), which was consistent with the results of our anti-inflammatory experiment. The glycosidic derivatives of kaempferol isolated from *T. chinense* can inhibit the expression of TNF-α, IL-6, IL-1β and PGE2, improve pulmonary edema *in vivo*, and inhibit the phosphorylation of NFκB and MAP kinase in mice *in vitro* (Sun et al. 2019). In conclusion, the extract of *T. chinense* has a good anti-inflammatory effect, and has the potential to be an inhibitor of IL6 and TNF-α.

Bacterial co-infection is a common complication of many viral respiratory tract infections. Viral infections alter the bacterial community in the upper respiratory tract, which may increase susceptibility to secondary infections and disease severity. This often leads to more severe clinical symptoms and significantly increases complication rates and mortality (Gupta et al. 2008; Rattanaburi et al. 2022). Bacterial co-infection is an important factor in almost all influenza deaths. Studies have found that *Staphylococcus aureus* and *Mycoplasma pneumoniae* have high infection rates in mild COVID-19 cases, and patients hospitalized for a long time are more likely to experience bacterial co-infection. Early diagnosis and treatment of bacterial co-infection can reduce the severity of COVID-19 and avoid complications (Davies-Bolorunduro et al. 2022). *T. chinense* is known as ‘natural antibiotic’ and has an excellent effect on treating inflammation, microbial infection and upper respiratory tract disease (Li et al. 2021). At the same time, *T. chinense* also has a broad-spectrum antibacterial effect, significantly inhibiting *S. aureus*, *Aeromonas hydrophila*, *Sarcina lutea*, *Bacillus cereus*, *Bacillus subtilis*, *Pseudomonas aeruginosa* (Liu et al. 2006). Therefore, it is not difficult to see that *T. chinense* can inhibit multiple bacterial infections, and has a high utilization prospect for bacterial co-infection. Based on this, we further investigated the anti-inflammatory and antiviral effects of *T. chinense.* The results showed that the ethanol extract of *T. chinense* significantly inhibited the replication and entry of SARS-CoV-2 in Vero cells and significantly reduced the levels of IL-6 and TNF-α produced by LPS-stimulated RAW264.7 cells. Therefore, the COVID-19 caused by SARS-CoV-2, *T. chinense* can not only significantly reduce viral load and inhibit the activity of a variety of bacteria, but also reduce the influence of inflammation caused by viral or bacterial infection. *T. chinense* is a natural antiviral medicine with great potential.

## Conclusions

The extracts of *T. chinense* significantly inhibited the replication and entry of SARS-CoV-2, had good anti-inflammatory effects, and inhibited the expression of the inflammatory factors IL-6 and TNF-α. The petroleum ether fractions, ethyl acetate fractions and *n*-butanol fractions have certain antiviral replication activity, but also have certain cytotoxicity. In the anti-inflammatory effect, the petroleum ether fraction, ethyl acetate fraction and *n*-butanol fraction showed obvious concentration dependence, and the expression levels of IL-6 and TNF-α induced by LPS gradually decreased with increasing concentration. The aqueous extract had a slight inhibitory effect on LPS-induced TNF-α expression only at the low concentration of 160 μg/mL. It is not difficult to suggest that *T. chinense* has good antiviral and anti-inflammatory activities, and it can be a potential candidate to fight SARS-CoV-2.

However, this experiment also has some limitations. Although *T. chinense* has strong antiviral activity, its cytotoxicity is also relatively high. If the separation of monomers can be considered in the subsequent experiments, then in-depth studies can be carried out to further achieve the purpose of increasing efficacy and reducing toxicity through the optimization of monomer chemical structure. In terms of the exploration of the mechanism of action, it is not possible to describe the concrete mechanism of *T. chinense* antiviral action. Therefore, it is possible to explore the specific pathway through which *T. chinense* exerts its antiviral effect and explore its mechanism of action in more detail.

## References

[CIT0001] Attallah NGM, El-Kadem AH, Negm WA, Elekhnawy E, El-Masry TA, Elmongy EI, Altwaijry N, Alanazi AS, Al-Hamoud GA, Ragab AE. 2021. Promising antiviral activity of *Agrimonia pilosa* phytochemicals against severe acute respiratory syndrome coronavirus 2 supported with *in vivo* mice study. Pharmaceuticals-Base. 14(12):1313. doi: 10.3390/ph14121313. PMC8709118 34959713

[CIT0002] Chen K, Chen H. 2020. Traditional Chinese medicine for combating COVID-19. Front Med. 14(5):529–532. doi: 10.1007/s11684-020-0802-9. 32705405 PMC7376532

[CIT0003] Cheng PC, Wong G. 1996. Honey bee propolis: prospects in medicine. Bee World. 77(1):8–15. doi: 10.1080/0005772X.1996.11099278.

[CIT0004] Chen WQ, Wang S, You QH. 2021. Application of LeDock and PyMOL software in the course teaching of natural product chemistry: a case study of traditional Chinese medicine in treating novel coronavirus pneumonia (COVID-19). Chem Educ. 42(24):97–100.

[CIT0005] Cinatl J, Morgenstern B, Bauer G, Chandra P, Rabenau H, Doerr HW. 2003. Glycyrrhizin, an active component of liquorice roots, and replication of SARS-associated coronavirus. Lancet. 361(9374):2045–2046. doi: 10.1016/s0140-6736(03)13615-x. 12814717 PMC7112442

[CIT0006] Davies-Bolorunduro OF, Fowora MA, Amoo OS, Adeniji E, Osuolale KA, Oladele O, Onuigbo TI, Obi JC, Oraegbu J, Ogundepo O, et al. 2022. Evaluation of respiratory tract bacterial co-infections in SARS-CoV-2 patients with mild or asymptomatic infection in Lagos, Nigeria. Bull Natl Res Cent. 46(1):115. doi: 10.1186/s42269-022-00811-2. 35469122 PMC9022018

[CIT0007] De Clercq E. 2019. New nucleoside analogues for the treatment of hemorrhagic fever virus infections. Chem Asian J. 14(22):3962–3968. doi: 10.1002/asia.201900841. 31389664 PMC7159701

[CIT0008] Del Valle DM, Kim-Schulze S, Huang HH, Beckmann ND, Nirenberg S, Wang B, Lavin Y, Swartz TH, Madduri D, Stock A, et al. 2020. An inflammatory cytokine signature predicts COVID-19 severity and survival. Nat Med. 26(10):1636–1643. doi: 10.1038/s41591-020-1051-9. 32839624 PMC7869028

[CIT0009] Dorner T, Kay J. 2015. Biosimilars in rheumatology: current perspectives and lessons learnt. Nat Rev Rheumatol. 11(12):713–724. doi: 10.1038/nrrheum.2015.110. 26282080

[CIT0010] El-Hilaly J, Amarouch MY, Morel N, Lyoussi B, Quetin-Leclercq J. 2021. *Ajuga iva* water extract antihypertensive effect on stroke-prone spontaneously hypertensive rats, vasorelaxant effects *ex vivo* and *in vitro* activity of fractions. J Ethnopharmacol. 270:113791. doi: 10.1016/j.jep.2021.113791. 33444718

[CIT0011] Focosi D, Maggi F, McConnell S, Casadevall A. 2022. Very low levels of remdesivir resistance in SARS-COV-2 genomes after 18 months of massive usage during the COVID19 pandemic: A GISAID exploratory analysis. Antiviral Res. 198:105247. doi: 10.1016/j.antiviral.2022.105247. 35033572 PMC8755559

[CIT0012] Fung TS, Liu DX. 2019. Human Coronavirus: host-pathogen interaction. Annu Rev Microbiol. 73(1):529–557. doi: 10.1146/annurev-micro-020518-115759. 31226023

[CIT0013] Gandhi S, Klein J, Robertson A, Pena-Hernandez MA, Lin MJ, Roychoudhury P, Lu PW, Fournier J, Ferguson D, Bakhash SAM, et al. 2022. De novo emergence of a remdesivir resistance mutation during treatment of persistent SARS-CoV-2 infection in an immunocompromised patient: a case report. Nat Commun. 13(1):1547. doi: 10.1038/s41467-022-29104-y. 35301314 PMC8930970

[CIT0014] Gupta RK, George R, Nguyen-Van-Tam JS. 2008. Bacterial pneumonia and pandemic influenza planning. Emerg Infect Dis. 14(8):1187–1192. doi: 10.3201/eid1408.070751. 18680640 PMC2600366

[CIT0015] Kalliolias GD, Ivashkiv LB. 2016. TNF biology, pathogenic mechanisms and emerging therapeutic strategies. Nat Rev Rheumatol. 12(1):49–62. doi: 10.1038/nrrheum.2015.169. 26656660 PMC4809675

[CIT0016] Kerber R, Lorenz E, Duraffour S, Sissoko D, Rudolf M, Jaeger A, Cisse SD, Camara AM, Miranda O, Castro CM, et al. 2019. Laboratory findings, compassionate use of favipiravir, and outcome in patients with *Ebola* virus disease, Guinea, 2015-A retrospective observational study. J Infect Dis. 220(2):195–202. doi: 10.1093/infdis/jiz078. 30788508 PMC6581890

[CIT0017] Kirtipal N, Bharadwaj S, Kang SG. 2020. From SARS to SARS-CoV-2, insights on structure, pathogenicity and immunity aspects of pandemic human coronaviruses. Infect Genet Evol. 85:104502. doi: 10.1016/j.meegid.2020.104502. 32798769 PMC7425554

[CIT0018] Liao Q, Chen Z, Tao Y, Zhang B, Wu X, Yang L, Wang Q, Wang Z. 2021. An integrated method for optimized identification of effective natural inhibitors against SARS-CoV-2 3CLpro. Sci Rep. 11(1):22796. doi: 10.1038/s41598-021-02266-3. 34815498 PMC8611036

[CIT0019] Li GH, Fang KL, Yang K, Cheng XP, Wang XN, Shen T, Lou HX. 2021. *Thesium chinense* Turcz.: An ethnomedical, phytochemical and pharmacological review. J Ethnopharmacol. 273:113950. doi: 10.1016/j.jep.2021.113950. 33610713

[CIT0020] Liu C, Li XT, Cheng RR, Han ZZ, Yang L, Song ZC, Wang ZT. 2018. Anti-oral common pathogenic bacterial active acetylenic acids from *Thesium chinense* Turcz. J Nat Med. 72(2):433–438. doi: 10.1007/s11418-018-1180-3. 29435792

[CIT0021] Liu YS, Pan L, Qi KZ, Jiang LK. 2006. Sensitivity test of effective extracts of Thesium chinense Turcz to seven types of bacteria. Guizhou Pharm. 06:564–566.

[CIT0022] Liu H, Ye F, Sun Q, Liang H, Li C, Li S, Lu R, Huang B, Tan W, Lai L. 2021. *Scutellaria baicalensis* extract and baicalein inhibit replication of SARS-CoV-2 and its 3C-like protease *in vitro*. J Enzyme Inhib Med Chem. 36(1):497–503. doi: 10.1080/14756366.2021.1873977. 33491508 PMC7850424

[CIT0023] Li X, Xu ZX, Ming YL. 2020. Application of Chinese herbal medicine in the prevention and treatment of COVID-19. Subtro Plant Sci. 49(02):83–92.

[CIT0024] Mo Y, Fisher D. 2016. A review of treatment modalities for Middle East Respiratory Syndrome. J Antimicrob Chemother. 71(12):3340–3350. doi: 10.1093/jac/dkw338. 27585965 PMC7109760

[CIT0025] Muller MP, Dresser L, Raboud J, McGeer A, Rea E, Richardson SE, Mazzulli T, Loeb M, Louie M, Canadian SRN; Canadian SARS Research Network. 2007. Adverse events associated with high-dose ribavirin: evidence from the Toronto outbreak of severe acute respiratory syndrome. Pharmacotherapy. 27(4):494–503. doi: 10.1592/phco.27.4.494. 17381375 PMC7168122

[CIT0026] Pan B, Fang S, Zhang J, Pan Y, Liu H, Wang Y, Li M, Liu L. 2020. Chinese herbal compounds against SARS-CoV-2: puerarin and quercetin impair the binding of viral S-protein to ACE2 receptor. Comput Struct Biotechnol J. 18:3518–3527. doi: 10.1016/j.csbj.2020.11.010. 33200026 PMC7657012

[CIT0027] Parveen Z, Deng Y, Saeed MK, Dai R, Ahamad W, Yu YH. 2007. Antiinflammatory and analgesic activities of *Thesium chinense* Turcz extracts and its major flavonoids, kaempferol and kaempferol-3-*O*-glucoside. Yakugaku Zasshi. 127(8):1275–1279. doi: 10.1248/yakushi.127.1275. 17666881

[CIT0028] Peng Y, Du N, Lei Y, Dorje S, Qi J, Luo T, Gao GF, Song H. 2020. Structures of the SARS-CoV-2 nucleocapsid and their perspectives for drug design. Embo J. 39(20):e105938. 32914439 10.15252/embj.2020105938 PMC7560215

[CIT0029] Pruijssers AJ, George AS, Schafer A, Leist SR, Gralinksi LE, Dinnon KH, 3rd, Yount BL, Agostini ML, Stevens LJ, Chappell JD, et al. 2020. Remdesivir Inhibits SARS-CoV-2 in human lung cells and Chimeric SARS-CoV expressing the SARS-CoV-2 RNA polymerase in mice. Cell Rep. 32(3):107940. doi: 10.1016/j.celrep.2020.107940. 32668216 PMC7340027

[CIT0030] Pum A, Ennemoser M, Adage T, Kungl AJ. 2021. Cytokines and chemokines in SARS-CoV-2 infections-therapeutic strategies targeting cytokine storm. Biomolecules. 11(1):91. doi: 10.3390/biom11010091. 33445810 PMC7828218

[CIT0031] Rattanaburi S, Sawaswong V, Chitcharoen S, Sivapornnukul P, Nimsamer P, Suntronwong N, Puenpa J, Poovorawan Y, Payungporn S. 2022. Bacterial microbiota in upper respiratory tract of COVID-19 and influenza patients. Exp Biol Med (Maywood). 247(5):409–415. doi: 10.1177/15353702211057473. 34775842 PMC8919321

[CIT0032] Rossi JF, Lu ZY, Jourdan M, Klein B. 2015. Interleukin-6 as a therapeutic target. Clin Cancer Res. 21(6):1248–1257. doi: 10.1158/1078-0432.CCR-14-2291. 25589616

[CIT0033] Shao L, Sun Y, Liang J, Li M, Li X. 2020. Decolorization affects the structural characteristics and antioxidant activity of polysaccharides from *Thesium chinense* Turcz: comparison of activated carbon and hydrogen peroxide decolorization. Int J Biol Macromol. 155:1084–1091. doi: 10.1016/j.ijbiomac.2019.11.074. 31715240

[CIT0034] Sun Z, Li Q, Hou R, Sun H, Tang Q, Wang H, Hao Z, Kang S, Xu T, Wu S. 2019. Kaempferol-3-*O*-glucorhamnoside inhibits inflammatory responses via MAPK and NF-kappaB pathways *in vitro* and *in vivo*. Toxicol Appl Pharmacol. 364:22–28. doi: 10.1016/j.taap.2018.12.008. 30528763

[CIT0035] Wang Z, Wang N, Yang L, Song XQ. 2022. Bioactive natural products in COVID-19 therapy. Front Pharmacol. 13:926507. doi: 10.3389/fphar.2022.926507. 36059994 PMC9438897

[CIT0036] Wang Z, Yang L. 2022. In the age of Omicron variant: paxlovid raises new hopes of COVID-19 recovery. J Med Virol. 94(5):1766–1767. doi: 10.1002/jmv.27540. 34936106

[CIT0037] Wang ZL, Yang LY. 2021. Chinese herbal medicine: fighting SARS-CoV-2 infection on all fronts. J Ethnopharmacol. 270:113869. doi: 10.1016/j.jep.2021.113869. 33485973 PMC7825841

[CIT0038] Wang Z, Yang L, Song XQ. 2022. Oral GS-441524 derivatives: next-generation inhibitors of SARS-CoV-2 RNA-dependent RNA polymerase. Front Immunol. 13:1015355. doi: 10.3389/fimmu.2022.1015355. 36561747 PMC9763260

[CIT0039] Wang Y, Zhang D, Du G, Du R, Zhao J, Jin Y, Fu S, Gao L, Cheng Z, Lu Q, et al. 2020. Remdesivir in adults with severe COVID-19: a randomised, double-blind, placebo-controlled, multicentre trial. Lancet. 395(10236):1569–1578. doi: 10.1016/S0140-6736(20)31022-9. 32423584 PMC7190303

[CIT0040] Westblade LF, Simon MS, Satlin MJ. 2021. Bacterial coinfections in Coronavirus disease 2019. Trends Microbiol. 29(10):930–941. doi: 10.1016/j.tim.2021.03.018. 33934980 PMC8026275

[CIT0041] Williamson BN, Feldmann F, Schwarz B, Meade-White K, Porter DP, Schulz J, van Doremalen N, Leighton I, Yinda CK, Perez-Perez L, et al. 2020. Clinical benefit of remdesivir in rhesus macaques infected with SARS-CoV-2. Nature. 585(7824):273–276. doi: 10.1038/s41586-020-2423-5. 32516797 PMC7486271

[CIT0042] Xu X, Han M, Li T, Sun W, Wang D, Fu B, Zhou Y, Zheng X, Yang Y, Li X, et al. 2020. Effective treatment of severe COVID-19 patients with tocilizumab. Proc Natl Acad Sci U S A. 117(20):10970–10975. doi: 10.1073/pnas.2005615117. 32350134 PMC7245089

[CIT0043] Yang L, Li Y-T, Miao J, Wang L, Fu H, Li Q, Wen W-B, Zhang Z-Y, Song R-W, Liu X-G, et al. 2020. Network pharmacology studies on the effect of Chai-Ling decoction in coronavirus disease 2019. Trad Chinese Med. 5:145–159.

[CIT0044] Yang L, Wang Z. 2021. Natural products, alone or in combination with FDA-approved drugs, to treat COVID-19 and lung cancer. Biomedicines. 9(6):689. doi: 10.3390/biomedicines9060689. 34207313 PMC8234041

[CIT0045] Zheng M, Karki R, Williams EP, Yang D, Fitzpatrick E, Vogel P, Jonsson CB, Kanneganti TD. 2021. TLR2 senses the SARS-CoV-2 envelope protein to produce inflammatory cytokines. Nat Immunol. 22(7):829–838. doi: 10.1038/s41590-021-00937-x. 33963333 PMC8882317

